# Immunoassay for Natamycin Trace Screening: Bread, Wine and Other Edibles Analysis

**DOI:** 10.3390/bios12070493

**Published:** 2022-07-06

**Authors:** Maksim A. Burkin, Anastasia G. Moshcheva, Inna A. Galvidis

**Affiliations:** 1 I.I. Mechnikov Research Institute for Vaccines and Sera, 105064 Moscow, Russia; ayyi@fmap.me (A.G.M.); galvidis@yandex.ru (I.A.G.); 2 A.P. Nelyubin Institute of Pharmacy, I.M. Sechenov First Moscow State Medical University, 119991 Moscow, Russia

**Keywords:** natamycin, food preservative E235, polyene antibiotics, hapten design, immunoassay, food safety

## Abstract

The antifungal drug natamycin (NAT) is widely used in medicine and in the food industry as preservative E235 for a wide variety of foods. The risk of the development of resistance to NAT and its spread in relation to other polyene antibiotics is fraught with the emergence of incurable infections. This work is devoted to the development of an immunoassay to investigate the prevalence of NAT use for food preservation. Two immunogen designs based on tetanus toxoid, conjugated to NAT through different sites of hapten molecules, were compared in antibody generation. Assay formats using heterologous coating antigens were superior for both antibodies. The ELISA variant demonstrated the highest sensitivity (IC_50_ = 0.12 ng/mL), and a limit of detection of 0.02 ng/mL was selected for NAT determination. The optimized extraction procedure provided a recovery rate of 72–106% for various food matrixes with variations below 12%. Cyclodextrins, as well as NAT–cyclodextrin complex formulations, showed no interference with the quantification of NAT. One hundred and six food product brands, including baked goods, wines, beers, drinks, sauces, and yogurts, were tested to assess the prevalence of the undeclared use of NAT as a preservative. The screening examination revealed three positive yogurts with an undeclared NAT incorporation of 1.1–9.3 mg/kg.

## 1. Introduction

Natamycin (NAT) is a polyene macrolide derived from *Streptomyces natalensis.* NAT, initially named pimaricin, was discovered by Struyk in 1955 in Pietermaritzburg (South Africa) in the culture of soil *Actinomycetes* isolates [1]. A wide spectrum of antifungal activity (*Candida*, *Aspergillus*, *Fusarium*, *Penicillium*, *Cephalosporium* and other species) is based on the mechanism of NAT action and its high affinity for binding to ergosterol, a target in the fungal membrane with the formation of a sponge-like polyene–ergosterol complex [2]. The resulting membrane damage results in a detrimental loss of essential ions and the inhibition of yeast and mold growth.

Due to this effect, NAT has found its application as an antimycotic agent in the treatment of fungal infections. However, its very limited solubility makes NAT suitable mainly for the treatment of topical infections such as blepharitis, conjunctivitis, and keratitis caused by susceptible fungi [3,4]. Fungal infections (mainly *Candida* fungi) of the skin and mucous surfaces provoked after therapy with antibiotics, corticosteroids or cytostatics, for example, skin and nail candidiasis, intestinal candidiasis, vaginitis, vulvitis, vulvovaginitis, balanoposthitis, ringworm, otomycosis, and otitis externa, can also be effectively treated with NAT [5,6,7,8].

Moreover, NAT is used as a food preservative against the growth of mold and yeast [9]. As a low toxic compound without odor and taste, NAT has been approved in many countries and has been preserved for more than 30 years as a preservative additive (E 235) for cheeses, sausages, yogurts, juices, and wines [10]. The low solubility of NAT has proven to be an advantage for protecting food surfaces without a significant diffusion into their depths. This preservative is usually effective at concentrations between 1 and 10 µg/mL [11]. Additionally, the antimycotic effect of NAT can be utilized as a coating material or incorporated into packaging material to increase a food product’s shelf life [12,13]. In order to regulate the use of this food additive, the European Commission has set a limit on the surface treatment of cheeses and sausages with NAT at a content of no more than 1 mg/kg in the final product [14]. As a result, the use of NAT by the food industry for the surface treatment of solid foods (cheeses, sausages) is considered to be safe, since the measured NAT concentrations are much lower than the acceptable daily intake level (ADI = 0.3 mg/kg body weight) [15]. However, a much higher intake of soluble NAT formulations (cyclodextrin–NAT complex) [16] utilized in beverages (juice, wine) and yoghurts can place pressure on resident human flora (fecal *Candida* spp.), reducing its susceptibility to NAT. Therefore, there are concerns that the developed resistance can transfer horizontally to pathogenic microflora and manifest itself in relation to other polyene antimycotics [15].

Baked products, which make up a significant portion of the human diet, are particularly susceptible to mold damage after baking, and thus can also be protected with NAT. Thus, NAT is approved in the USA for the treatment of bread, tortillas, cakes, and muffins up to 7–20 mg/kg. In China, NAT suspensions at 200 ± 300 mg/kg concentration can be used for spraying or dipping bakery products, ensuring that the residual content in the product does not exceed 10 mg/kg [11].

The present study focuses on the development of an antibody-based screening method to detect NAT in high-consumption foods and to assess the prevalence of NAT use for food preservation.

## 2. Methods

### 2.1. Chemicals

Natamycin (NAT) was kindly provided by the Gause Institute of New Antibiotics (Moscow, Russia). Complete (CFA) and incomplete (IFA) Freund adjuvants, adipic acid dihydrazide (ADH), ε-aminocapronic acid (ACA), N-hydroxysuccinimide (NHS), 1-ethyl-3-(3-dimethylaminopropyl) carbodiimide (EDC), sodium periodate (PI), sodium borohydride, dimethylsulfoxide (DMSO), dimethylformamide (DMF), cyclodextrines, αCD, βCD, methyl-βCD (MeβCD), and 2 hydroxypropyl-βCD (HP-βCD) were obtained from Sigma-Aldrich (Saint Louis, MO, USA). Tetanus toxoid (TTd) was provided from Microgen (Moscow, Russia). Gelatin (Gel) was from Bio-Rad (Hercules, CA, USA); 3,5,3′,5′-tetramethylbenzidine (TMB)-containing substrate mixture was provided by Bioservice Ltd. (Moscow, Russia); goat anti-rabbit IgG antibodies conjugated to horseradish peroxidase (GAR-HRP) were from IMTEK (Moscow, Russia). 

### 2.2. Synthesis of Immunogens and Coating Antigens

*TTd-adh-NAT and TTd-aca-NAT.*

EDC (7.68 mg, 40 µmol) and NHS (4.6 mg, 10 µmol) powders were dissolved in a TTd solution (6.0 mg, 40 nmol) of 1.15 mL and stirred for 30 min at RT. The resultant solution of protein with activated carboxyl groups was divided into two equal volumes. One portion was supplemented with 300 equivalents of ADH (1.05 mg, 6 µmol) in a 0.5 mL 0.05 M carbonate buffer (CB, pH 9.5). The second portion was combined with 300 eqs of ACA (0.78 mg, 6 µmol) in a 0.5 mL CB. The mixtures were stirred for 2 h at RT and then dialyzed overnight against 5L of water.

NAT (666 µg, 1 µmol) from 5 mg/mL solution in DMSO was added to EDC (384 µg, 2 µmol) and NHS (230 µg, 2 µmol), both in 10 mg/mL of DMF and stirred for 1 h. Then, the activated NAT was dropwise added to a 1.0 mL solution of TTd-adh (3 mg, 20 nmol) adjusted to pH 9.0 using Na_2_CO_3_. Two hours of stirring at RT were conducted to form TTd-adh-NAT conjugate. 

The carboxylated protein TTd-aca (3 mg, 20 nmol) in 1.0 mL H_2_O was again activated with EDC (7.68 mg, 40 µmol) and NHS (4.6 mg, 10 µmol) for 30 min and 50 eqs of NAT (666 µg, 1 µmol) in 0.25 mL CB was added. The conjugation was continued for 2 h to obtain TTd-aca-NAT. The resultant conjugates were purified from excessive hapten using overnight dialysis against 2 × 1 L of 10% DMSO and 5 L of water. The prepared conjugates were used as immunogens.

*Gel-NAT(ae) and Gel(pi)-NAT.*

NAT was activated using EDC/NHS as described above. The activated hapten was dropwise added to a Gel solution in CB. The ratio between Gel and NAT was taken as 1/10 and 1/30.

Gel (8 mg, 50 nmol) in 0.8 mL of water was accomplished with 0.2 mL of sodium periodate (1.0 mg, 5 µmol) and stirred for 20 min. After an overnight dialysis at 4 °C against 5 L of distilled water, the oxidized Gel (4 mg, 0.5 mL) reacted with 10 or 30 molar equivalents of NAT in CB. After 2 h of stirring at RT, sodium borohydride (50 µL, 4 mg/mL) was added and stirred for 1 h. To remove excessive reagents, dialysis against 10% DMSO and distilled water was carried out. 

All dialysates were supplemented with glycerol and stored as 1 mg/mL solutions at –20 °C. The prepared TTd conjugates were used as immunogens, and the Gel-based conjugates were served as coating antigens.

### 2.3. Immunization Procedure and Antibody Preparation

Rabbits (2.0–2.5 kg) were obtained from the Scientific and Production Centre for Biomedical Technologies (Elektrogorsk, Russia). The animal experiments were approved by the Ethics Committee of the I. Mechnikov Research Institute for Vaccines and Sera and performed in accordance with the guidelines for the care and use of laboratory animals in biomedical research. 

TTd-adh-NAT and TTd-aca-NAT emulsified in CFA were subcutaneously injected in multiple points on the backs of the rabbits. The first immunization and booster injections (in IFA) were carried out with 100 μg of immunogens. In the subsequent administration of immunogens, the doses were gradually reduced from 100 to 50 μg. Each monthly booster was followed in a week by a blood sample collection for immune response monitoring. Glycerol-supplemented antisera samples were stored at −20 °C and used as antibody preparations.

### 2.4. ELISA Optimization

The interaction of antisera with antigens coated on 96-well polystyrene Costar plates was studied in indirect ELISA with an antibody titer and the sensitivity of NAT determination was expressed in half-inhibition concentration (IC_50_) according to the usual procedure [17]. The conjugates Gel-NAT(ae) and Gel(pi)-NAT taken as 0.1–3 μg/mL solutions in CB were coated in the plates for 16 h at 4 °C. The plates were washed 3 times with phosphate-buffered saline containing 0.05% Tween 20 (PBS-T) and were filled with 0.1 mL of antisera serially diluted in PBS-T with 1% BSA and 0.1 mL PBS-T (B_0_) or NAT standard solutions (0.01–1000 ng/mL, B). The incubation of the plates occurred for 1 h at 25 °C. After the washing, 0.1 mL of GAR-HRP was added to detect bound antibodies and was incubated for 1 h at 37 °C. The substrate solution containing TMB was added 0.1 mL/well. The enzymatic reaction was terminated 30 min later with 0.5 M sulfuric acid (0.1 mL) and the colored reaction product was registered at 450 nm using a LisaScan reader (Erba Manheim, Czech Republic).

The working antibody titer was determined as an antibody dilution which provided a B_0_ value within the 0.8–1.2 range. The optimized parameters were considered in relation to the ratio of immunoreagents providing the highest sensitivity of NAT determination, i.e., the lowest IC_50_ index. The relative antibody binding (B/B_0_, %) vs. analyte concentration plots served as standard curves constructed by using a four-parameter logistic fitting in GraphPad Prism 8.0 software. The dynamic range of the assay was accepted as IC_20_–IC_80_ and the limit of detection (LOD) was calculated as B_0_-3 × SD.

The specificity was estimated by the cross-reactivity (CR) of related polyenes and aminoglycoside-bearing antibacterials [18] as the percentage IC_50 NAT_/IC_50 ANALOG._

### 2.5. Sample Collection, Pretreatment and Analysis

Food samples for the analysis were purchased from local retail outlets. To study the prevalence of NAT food preservation, sampling followed the principle of the maximum diversity of product types and/or their manufacturers. 

*Bakery products.* The surface layer of the product up to 5 mm was cut off, dried to a rusk state, then crushed into crumbs and stored in the dark at room temperature until analysis. One-gram weighed portions were poured with 5 mL of methanol (MeOH) and periodically vortexed vigorously. The resulting extracts were diluted appropriately with a buffer and analyzed by ELISA.

*Wines.* Aliquot samples of 10 mL were frozen at −20 °C and stored until analysis. After thawing and the separation of wine sediment, the supernatant was diluted with MeOH 5 times and vigorously stirred. Extracts diluted with PBST were analyzed by ELISA. 

*Beers, Juices, and Sauces.* Collected samples were stored as frozen aliquots until analysis or used within the shelf life. After a similar MeOH extraction, some ingredients in the matrix formed a precipitate which was removed by centrifugation for 5 min at 10,000 rpm. A PBST-diluted supernatant was analyzed by ELISA. 

*Yoghurts.* Purchased yoghurts were stored at 4C and tested within the stated expiration date. Thoroughly homogenized samples were taken (≈0.3 mL) using an insulin syringe without a needle, weighed in an Eppendorf tube (0.3 g) and supplemented with MeOH (1.5 mL). After extraction with vigorous vortexing, the samples were centrifuged for 5 min at 10,000 rpm to isolate the supernatant. Appropriately PBST-diluted supernatant was analyzed for NAT content.

*Cheeses.* One gram of grounded cheese rind and separate inner part (about 5 mm in depth) samples were extracted with 5 mL of MeOH for 20 min at 25 °C. The extract was vigorously vortexed and then centrifuged for 5 min at 10,000 rpm. Then, the supernatant diluted with PBST was tested by ELISA.

## 3. Results and Discussion

### 3.1. Immunogen Synthesis

**Figure 1:**
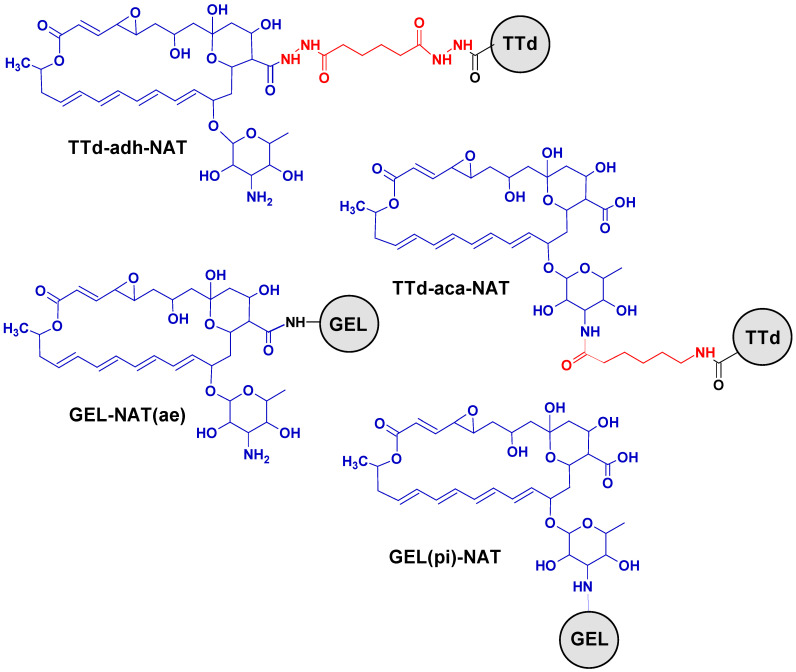
Structures of NAT-based immunogens and coating antigens for indirect competitive ELISA. NAT—blue; protein carrier—black; spacer—red.

**Figure 2:**
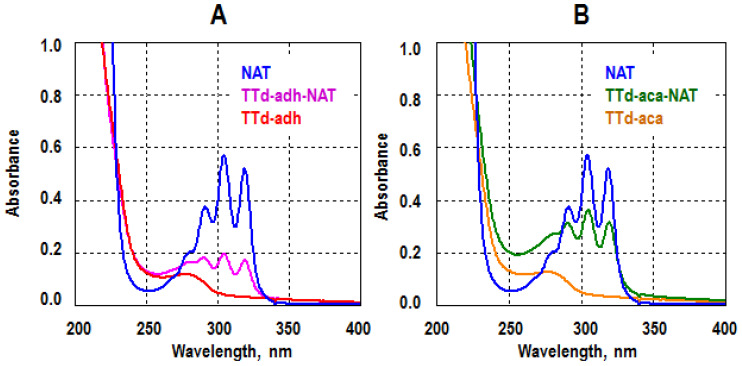
UV-Vis spectra of immunogen conjugates TTd-adh-NAT (**A**) and TTd-aca-NAT (**B**). Conjugates and modified TTd, 0.1 mg/mL; NAT, 10 µg/mL in water.

To obtain antibodies, a pair of NAT conjugates were prepared using TTd, a potent vaccine antigen and a carrier for various haptens [19]. The conjugation of TTd-adh-NAT and TTd-aca-NAT was directed to bond to different NAT sites, the carboxyl at position C25, and the mycosamine amine (Figure 1). For this, different linkers were used for the modifying of protein carriers. The ADH-modified TTd was linked to NAT carboxyl, while the ACA-modified TTd was suitable for conjugation through the NAT amine. The formation of conjugates was confirmed by their UV-spectra (Figure 2) which combined the features of both TTd and NAT. Despite an equal 50-fold excess of NAT in the carrier at the syntheses of these conjugates, the hapten load was 6.3 (mol/mol) for TTd-adh-NAT and 12.7 (mol/mol) for TTd-aca-NAT. The spectral changes of the coating Gel-based conjugates were also specific, but less pronounced due to the low excess of hapten during synthesis. 

### 3.2. Selection of Immunoreagents and Examination of Assay Specificity

**Figure 3:**
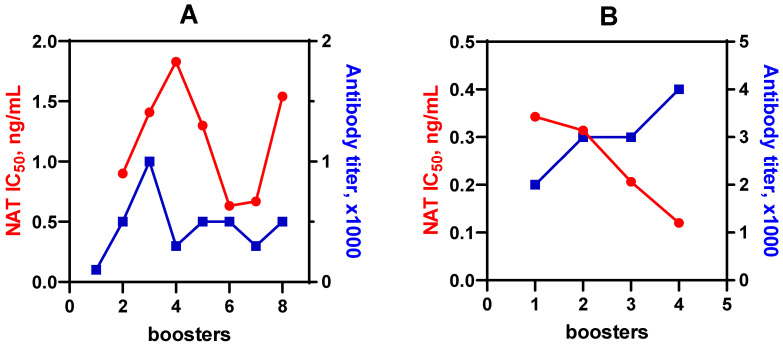
Dynamics of antibody titer and IC_50_ for NAT determination during immunization with TTd-adh-NAT (**A**) and TTd-aca-NAT (**B**). Coating antigens: Gel(pi)-NAT (**A**) and Gel-NAT(ae) (**B**). IC_50_ values were calculated from calibration curves based on standard triplicates. The titers are represented by the dilution factor of the antibody resulting in a binding absorbance of 0.8–1.2.

During the immunization course, blood samples were taken from rabbits to monitor the immune response. Maturing antibodies were analyzed by their binding activity (titers) and, more importantly, by the sensitivity (IC_50_) of NAT determination. In addition, different coating antigens were compared as factors affecting the assay sensitivity and specificity [20,21]. As a result of the comparison, it was found that the heterologous binding site of NAT to Gel made the coating antigens more preferable. Thus, the better pair of immunoreagents, namely, anti-TTd-adh-NAT–Gel(pi)-NAT and anti-TTd-aca-NAT–Gel-NAT(ae) were selected, and these interactions were optimized (Figure 3). 

The analysis of the antibody properties in terms of the dynamics of immune response revealed that the highest sensitivity (IC_50_ = 0.63 ng/mL) was provided by anti-TTd-adh-NAT #6. At the same time, the activity of interaction with the heterologous antigen was rather low (1/500) (Figure 3A). Anti-TTd-aca-NAT antibodies were generated against immunogens with a higher hapten load. Therefore, they demonstrated a higher binding activity with heterologous-oriented NAT on the coating antigen (#4-1/4000) (Figure 3B). The sensitivity of NAT determination improved along with immunization and achieved its best value after the fourth booster (IC_50_ = 0.12 ng/mL). 

A study of the specificity of these assays for the related polyene macrolides of amphotericin B and nystatin did not reveal any cross-reactions (Table 1). 

Thus, these studies allowed us to select the most preferred reagents for the NAT-specific assay, which turned out to be more than an order of magnitude more sensitive than in previous reports [22,23] and in our recent group-specific immunoassay for polyenes [24]. The comparative analytical characteristics of the developed immunoassays from the available literature are given in Table 2. 

### 3.3. Evaluation of NAT Determination in Its Formulations with Cyclodextrins

**Figure 4:**
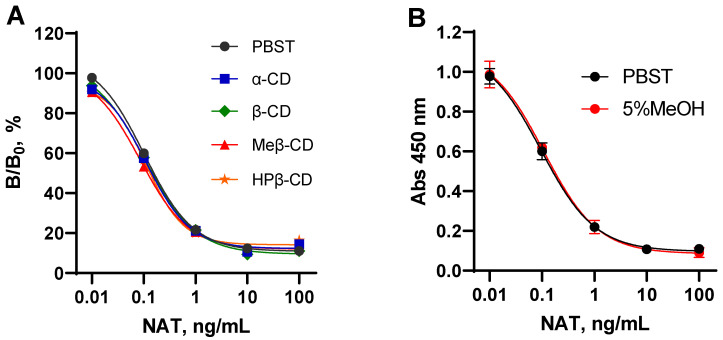
Effect of cyclodextrins (**A**) and methanol (**B**) on NAT standard curves in the developed ELISA. NAT standard curves were generated in CD solutions (10 µg/mL). Each point is presented by average (*n* = 4). The error bars correspond to the standard deviation.

Due to poor solubility, the use of NAT is limited to the surface treatment of solid products. The preservation of soft and liquid foods requires soluble formulations of NAT, which are more effective. Cyclodextrins (CDs) are known as solubility enhancers suitable and safe for food industry. They act as host molecules to form inclusion complexes with a wide variety of guest molecules. These properties of CD are used in NAT formulations for easy surface spraying or incorporation into liquid foods, such as wines, juices, yoghurts, etc. [16]. In addition, CDs themselves are widely used in the food industry as multifunctional stabilizers [25]. However, it has been reported that CDs may alter the measurement of guest molecules [26]. Therefore, it was necessary to find out whether CDs or CD–NAT complexes would interfere with the immunodetection of NAT. The effect of CDs as food additives in NAT determination was found to be negligible. The inhibition rate of NAT standards in CD solutions did not differ significantly from those in the assay buffer (Figure 4A). 

In addition, it is known that organic solvents destroy CD–guest molecule complexes [27]. Thus, due to the fact that the extraction of NAT from food matrices is usually carried out with methanol, the presence of CD-NAT complexes in the samples did not prevent the detection of NAT, since they were destroyed. 

### 3.4. Extraction Optimization and Recovery Experiments

The widespread use of NAT as a preservative necessitates the verification of the developed test for the analysis of various food products. Cheese and sausage treatments with NAT were found to be safe, despite the rather high usage level of 20 mg/kg [28]. In this regard, we primarily focused on the analysis of high-consumption products and those where high dietary exposure to NAT could be provoked by the use of soluble formulations of antibiotics. Here, bakery products were considered as high-consumption products, and the category of liquid products included wines, beers, juices, yoghurts and sauces.

The preliminary experiments on the distribution of NAT between immiscible systems of water/ethyl acetate and water/hexane resulted in 4–10-fold higher concentrations in water phases. Thus, these organic solvents were excluded from the pretreatment of samples as ineffective extractants. Comparative studies also allowed us to ensure that methanol was preferable to acetonitrile as an extractant. A number of reports regarding various analytical methods for the determination of NAT in various food matrices have also used methanol extraction [23,29,30]. These served as the bases for the choice of methanol as an extracting agent in the determination of NAT in the entire spectrum of the studied products. Methanol in extracts can affect immune binding; however, when its content was diminished to 5%, the effect on the standard curve was negligible (Figure 4B).

**Figure 5:**
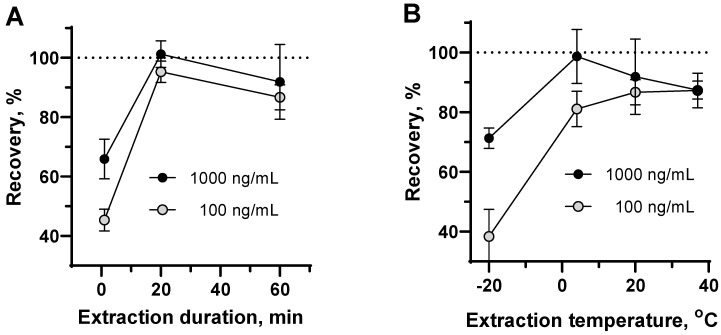
The influence of the duration and temperature of methanol extraction on NAT recovery from beer samples. The extraction procedure for samples fortified with NAT at 1000 and 100 ng/mL was carried out at 20 °C (**A**) and for 1 h (**B**); each point is presented by average and standard deviation (*n* = 4).

The efficacy of the extraction procedure was estimated using beer samples fortified with NAT. It was found that the completeness of NAT detection could depend on the duration of the extraction and temperature (Figure 5). 

The most suitable conditions for sample extractions were found to be 20 min at 20 °C. However, 1 h at 4 °C also proved to be an acceptable condition, while very fast and cold extraction was not effective. The adequacy of the optimized extraction procedure for other food products was illustrated by the NAT recovery rate in Table 3. 

It can be seen that the developed test makes it possible to determine NAT levels and even trace amounts that are orders of magnitude lower than the established threshold. The range of concentrations to be determined can be selected by the dilution factor of the sample extract. Thus, 1–10 µg/kg or µg/L levels of NAT could be detected in solid and liquid products when the dilution of the extract was of 15–20 times. Higher levels of NAT 1000–100 µg/kg required a 200-fold extract dilution. The generalized recovery factor ranged from 72% to 106% with a variation of no more than 12%.

### 3.5. Determination of NAT in Real Products

Thirty different types of bakery products from regional manufacturers, including wheat and rye breads, loaves and baguettes, buns and flatbreads, sandwich breads and milk breads, ciabattas and pita breads, croissants and muffins were examined for traces of NAT in their surface layer. No positive samples were found, confirming the non-use of the NAT preservative in these types of products (Table S1). 

No NAT traces were revealed in different wine samples (*n* = 25) from nine countries in Europe, South America (*n* = 4) and South Africa (*n* = 2). All other tested beverages (*n* = 25), including fruit and vegetable juices, berry fruit drinks and beers, cider and kvass were free of NAT, as were soy sauce samples (*n* = 3) (Tables S2 and S3).

Among yoghurt samples (*n* = 24) without indications concerning preservation with NAT, three positive samples were revealed. The content of NAT in these samples was 1.08 ± 0.12, 4.93 ± 0.4, and 9.25 ± 0.8 mg/kg (Table S4).

Only the cheese samples (*n* = 5) were tested as positive samples since they were collected in accordance with the indication of their NAT treatment. The measurement of NAT in cheese rinds (1–2 mm) revealed a 0.3–19.7 mg/kg concentration, whereas the inner layer (>10 mm) from the same samples contained 0.11–2.8 mg/kg (Table S5). 

## 4. Conclusions

Two conjugate designs based on tetanus toxoid as a carrier protein and NAT linked through different sites of hapten molecules were considered as immunogens for antibody generation. The same sites of NAT conjugation were involved in the synthesis of coating antigens based on gelatin. The monitoring of immune responses allowed us to select the most mature antibodies. The resulting antibodies and coating antigens served as the basis for the ELISA variants that were compared. Heterologous assay formats were found to be superior for both antibodies, and the ELISA variant demonstrated a higher sensitivity (IC_50_ = 0.12 ng/mL) and a limit of detection of 0.02 ng/mL was selected as the final tool for NAT determination. The extraction procedure (solvent, duration, temperature) was optimized and provided a recovery rate of 72–106% for various food matrixes. Cyclodextrins, as well as NAT–cyclodextrin complexes, were tested as soluble NAT formulations and showed no interference with NAT quantitation. One hundred and six food brands were studied, including baked goods, wines, beers, drinks, sauces, and yoghurts, to estimate the prevalence of the illegal (undeclared) use of NAT as a preservative. Only three yoghurts tested showed undeclared NAT incorporation at 1.1–9.3 mg/kg. In the cheese samples with indicated NAT treatments, the NAT content in both the rind and the inner layer was confirmed by the developed test to be in the range of 0.3–19.7 mg/kg and 0.11–2.8 mg/kg, respectively.

## Figures and Tables

**Table 1:** Specificity of two selected ELISA variants.

AGs	ELISA Variants Based on “Antibody Coating Antigens” Reagents			
	Anti-TTd-adh-NAT–Gel(pi)-NAT		Anti-TTd-aca-NAT–Gel-NAT(ae)	
	IC_50_, ng/mL	CR, %	IC_50_, ng/mL	CR, %
NAT	0.63	100	0.12	100
Amphotericin B	>1000	<0.1	>1000	<0.01
Nystatin	>1000	<0.1	>1000	<0.01

**Table 2:** Analytical parameters of NAT determination in the developed immunoassays.

Immuno Assays	Antibody, Species	Immunogen, Coating Antigen	IC_50_, ng/mL	Working Range, ng/mL	LOD, ng/mL	Matrix	Reference
ELISA	McAb mouse	BSA-NAT(ga) OVA-NAT(ga)	1.69	0.64–4.46	0.59	Milk Juice Yoghurt Cheese	[21]
LFIA	McAb mouse	BSA-NAT(ga) OVA-NAT(ae)	nd	5–20	5.0 10.0	Milk Yoghurt	[22]
ELISA	PcAb rabbit	TTd-ATB(cuaac) GEL(pi)-NAT	6.0	0.6–50	0.1	Serum	[23]
ELISA	PcAb rabbit	TTd-aca-NAT Gel-NAT(ae)	0.12	0.04–1.2	0.02	Bakery products Wine and Beer Beverages Yoghurt Cheese	Present study

ELISA—enzyme-linked immunosorbent assay; LFIA—lateral flow immunoassay; McAb—monoclonal antibody; PcAb—polyclonal antibody; ATB—amphothericin B; GA—glutaraldehyde linker agent; AE—active ester reaction; CuAAC—copper-catalyzed azide–alkyne cycloaddition; PI—periodate oxidation; nd—not determined.

**Table 3:** Recovery of NAT from various food products and beverages using the developed ELISA.

Matrix	Fortification Level, µg/kg or µg/L	Extract Dilution	RC, %	CV, %
White bread	1000	200	102	10.2
(wheat)	100	200	98	10.9
	10	20	75.4	6.5
	1	20	89.4	5.7
Black bread	1000	200	81.6	10.3
(rye)	100	200	79.8	11.2
	10	20	88.7	10.1
	1	20	86.8	7.3
Wine	1000	200	96.0	8.3
(red dry)	100	200	84.4	3.1
Wine	1000	200	96.4	8.8
(white semisweet)	100	200	84.4	10.9
Beer	1000 100	200 200	101.2 95.3	4.5 3.6
Juice	1000	200	98.6	9.5
(apple)	100	200	91.7	9.2
Juice	1000	200	106	9.9
(banana)	100	200	94.0	10.0
Juice	1000	200	92.7	9.6
(orange)	100	200	89.6	9.1
Juice	1000	200	95.4	11.1
(tomato)	100	200	73.5	9.2
Juice	10	15	105	9.8
(pear)	1	15	94.4	12.0
Sauce	1000	200	85.8	4.3
(soy)	100	200	72.4	4.1
Yoghurt	100	200	87.5	7.9
	10	200	84.4	3.9
Cheese	100	200	91.8	6.1
	10	200	102.7	4.1

Recovery—RC; coefficient of variation—CV.

## Data Availability

The datasets used and/or analyzed during the current study are available from the corresponding author on request.

## Supplementary Materials

The following supporting information can be downloaded at: https://www.mdpi.com/article/10.3390/bios12070493/s1, Table S1–S5: The results of food products screening for NAT;
