# Supplementary material for: Immunoassay for Natamycin Trace Screening: Bread, Wine and Other Edibles Analysis
Source: Biosensors (Basel). 2022 Jul 6;12(7):493. doi: 10.3390/bios12070493 (PMC9312873; doi:10.3390/bios12070493)
Supplement: Supplementary file 1 [file biosensors-12-00493-s001.zip › biosensors-1790174-supplementary.pdf]

Immunoassay for Natamycin Trace Screening: Bread, Wine and Other Edibles Analysis

Maksim A. Burkin [1,*], Anastasia G. Moshcheva [1,2] and Inna A. Galvidis [1]

[1] I.I. Mechnikov Research Institute for Vaccines and Sera, 105064 Moscow, Russia; ayyi@fmap.me (A.G.M.); galvidis@yandex.ru (I.A.G.)
[2] A.P. Nelyubin Institute of Pharmacy, I.M. Sechenov First Moscow State Medical University, 119991 Moscow, Russia
* Correspondence: burma68@yandex.ru; Tel./Fax: +7-495-9172753

Determination of NAT in real products

Table S1. The results of bakery products screening for NAT.

Sample number	Food products	Found concentration, ng/mL	Dilution factor	Final content, mg/kg or mg/L
Bakery products				
1	Wheat tortilla	< LOD	1000	< 0.02
2	Sandwich bread	< LOD	1000	< 0.02
3	Croissant 1	< LOD	1000	< 0.02
4	Milk bread	< LOD	1000	< 0.02
5	Ciabatta	< LOD	1000	< 0.02
6	Croissant French	< LOD	1000	< 0.02
7	Croissant with filling 1	< LOD	1000	< 0.02
8	Croissant with filling 2	< LOD	1000	< 0.02
9	Sweet bun	< LOD	1000	< 0.02
10	Wheat bread 1	< LOD	100	< 0.002
11	Baguette	< LOD	1000	< 0.02
12	Rye loaf 1	< LOD	100	< 0.002
13	Lithuanian bread	< LOD	1000	< 0.02
14	Pita Mediterranean	< LOD	1000	< 0.02
15	Fragrant bread	< LOD	1000	< 0.02
16	Pita	< LOD	1000	< 0.02
17	Wheat loaf	< LOD	1000	< 0.02
18	Chocolate cupcake	< LOD	1000	< 0.02
19	Cake with raisins 1	< LOD	1000	< 0.02
20	Rye loaf 2	< LOD	1000	< 0.02
21	Rye loaf 3	< LOD	1000	< 0.02
22	Croissant 2	< LOD	1000	< 0.02
23	Muffin	< LOD	1000	< 0.02
24	Muffin with filling	< LOD	1000	< 0.02
25	Pancake	< LOD	1000	< 0.02
26	Cake with raisins 2	< LOD	1000	< 0.02
27	Wheat bread 2	< LOD	1000	< 0.02
28	Custard bread 1	< LOD	1000	< 0.02
29	Grain bun	< LOD	1000	< 0.02
30	Custard bread 2	< LOD	1000	< 0.02

Table S2. The results of wine screening for NAT.

Sample number	Food products	Found concentration, ng/mL	Dilution factor	Final content, mg/kg or mg/L
Wines				
1	Red dry (Spain) 1	< LOD	1000	< 0.02
2	White dry (Germany) 1	< LOD	1000	< 0.02
3	Red dry (Italy) 1	< LOD	1000	< 0.02
4	Red semisweet (Russia)	< LOD	1000	< 0.02
5	Red dry (Italy) 2	< LOD	1000	< 0.02
6	Red dry (Spain) 2	< LOD	1000	< 0.02
7	Red dry (Spain) 3	< LOD	1000	< 0.02
8	Red dry (Spain) 4	< LOD	1000	< 0.02
9	Red dry (Chile) 1	< LOD	1000	< 0.02
10	Red sweet (Georgia)	< LOD	1000	< 0.02
11	Red dry (Spain) 5	< LOD	1000	< 0.02
12	Red semisweet (Georgia)	< LOD	1000	< 0.02
13	Red dry (Italy) 3	< LOD	1000	< 0.02
14	White dry (Austria)	< LOD	1000	< 0.02
15	Red dry (Chile) 2	< LOD	1000	< 0.02
16	White semisweet (Georgia)	< LOD	1000	< 0.02
17	Red dry (Italy) 4	< LOD	1000	< 0.02
18	White dry (Russia)	< LOD	1000	< 0.02
19	White brut (Italy)	< LOD	1000	< 0.02
20	White dry (Germany) 2	< LOD	1000	< 0.02
21	Red dry (South Africa) 1	< LOD	1000	< 0.02
22	Red dry (Argentina)	< LOD	1000	< 0.02
23	Red dry (Russia)	< LOD	1000	< 0.02
24	White dry (Argentina)	< LOD	1000	< 0.02
25	Red dry (South Africa) 2	< LOD	1000	< 0.02

Table S3. The results of beverages and sauces screening for NAT.

Sample number	Food products	Found concentration, ng/mL	Dilution factor	Final content, mg/kg or mg/L
Beverages				
1	Cheery juice 1	< LOD	1000	< 0.02
2	Banana juice 1	< LOD	1000	< 0.02
3	Briar juice	< LOD	1000	< 0.02
4	Banana juice 2	< LOD	1000	< 0.02
5	Wheat beer	< LOD	1000	< 0.02
6	Blanche	< LOD	1000	< 0.02
7	Lager	< LOD	1000	< 0.02
8	Mango cider	< LOD	1000	< 0.02
9	Orange juice	< LOD	1000	< 0.02
10	Cheery juice 2	< LOD	1000	< 0.02
11	Multifruit juice	< LOD	1000	< 0.02
12	Apple juice 1	< LOD	1000	< 0.02
13	India Pale Ale	< LOD	1000	< 0.02
14	Blackberry mead	< LOD	1000	< 0.02
15	Cherry cider	< LOD	1000	< 0.02

16	Beer	< LOD	1000	< 0.02
17	Maple porter	< LOD	1000	< 0.02
18	Mango juice	< LOD	1000	< 0.02
19	Banana juice	< LOD	1000	< 0.02
20	Orange juice	< LOD	1000	< 0.02
21	Apple juice 2	< LOD	1000	< 0.02
22	Tomato juice	< LOD	1000	< 0.02
23	Carrot juice	< LOD	1000	< 0.02
24	Pear juice	< LOD	60	< 0.0012
25	Kvass	< LOD	1000	< 0.02
Sauces				
1	Soy sauce 1	< LOD	1000	< 0.02
2	Soy sauce 2	< LOD	1000	< 0.02
3	Soy sauce 3	< LOD	1000	< 0.02

Table S4. The results of yoghurts screening for NAT.

Sample number	Food products	Found concentration, ng/mL	Dilution factor	Final content, mg/kg or mg/L
Yoghurts*				
1	Yoghurt drink	< LOD	1000	< 0.02
2	Semisolid yoghurt	< LOD	1000	< 0.02
3	Semisolid yoghurt	< LOD	1000	< 0.02
4	Greek yoghurt	< LOD	1000	< 0.02
5	Semisolid yoghurt	< LOD	1000	< 0.02
6	Semisolid yoghurt	< LOD	1000	< 0.02
7	Greek yoghurt	1.08 ± 0.12	1000	1.08 ± 0.12
8	Semisolid yoghurt	< LOD	1000	< 0.02
9	Thermostatic yoghurt	< LOD	1000	< 0.02
10	Yoghurt drink	< LOD	1000	< 0.02
11	Semisolid yoghurt	< LOD	1000	< 0.02
12	Yoghurt drink	< LOD	1000	< 0.02
13	Semisolid yoghurt	< LOD	1000	< 0.02
14	Semisolid yoghurt	< LOD	1000	< 0.02
15	Semisolid yoghurt	4.926 ± 0.406	1000	4.926 ± 0.406
16	Yoghurt drink	< LOD	1000	< 0.02
17	Semisolid yoghurt	< LOD	1000	< 0.02
18	Semisolid yoghurt	< LOD	1000	< 0.02
19	Greek yoghurt	< LOD	1000	< 0.02
20	Sheep milk yoghurt	< LOD	1000	< 0.02
21	Greek yoghurt	< LOD	1000	< 0.02
22	Yoghurt drink	< LOD	1000	< 0.02
23	Greek yoghurt	< LOD	1000	< 0.02
24	Greek yoghurt	9.251 ± 0.868	1000	9.251 ± 0.868

* NAT is not listed on the product label

Table S5. The results of cheeses screening for NAT.

Sample number	Food products	Found concentration, ng/mL		Dilution factor	Final content, mg/kg or mg/L	
Cheeses*		Rind	Inner layer		Rind	Inner layer
1	Semi-hard	1.223 ± 0.029	0.173 ± 0.02	1000	1.22 ± 0.03	0.17 ± 0.02
2	Semi-hard	0.287 ± 0.043	0.112 ± 0.033	1000	0.29 ± 0.04	0.11 ± 0.03
3	Hard	1.965 ± 0.135	0.281 ± 0.175	10000	19.7 ± 0.14	2.81 ± 0.18
4	Processed	0.322 ± 0.015	0.283 ± 0.019	1000	0.32 ± 0.02	0.28 ± 0.02
5	Hard	0.813 ± 0.102	0.593 ± 0.039	1000	0.81 ± 0.10	0.59 ± 0.04

* NAT is listed on the product label
